# Supplementary material for: Digital transformation and supply chain efficiency improvement: An empirical study from a-share listed companies in China
Source: PLoS One. 2024 Apr 16;19(4):e0302133. doi: 10.1371/journal.pone.0302133 (PMC11020941; doi:10.1371/journal.pone.0302133)
Supplement: Attachment — Submitted filename: Response to Reviewers8.docx [file pone.0302133.s001.docx]

Dear Dr. Ioana Gutu and Reviewers,

We are profoundly grateful for the opportunity to revise our manuscript and resubmit it for consideration for publication in PLOS ONE. We deeply appreciate the constructive feedback provided by the academic editor and reviewers. Their insights have been invaluable in enhancing the quality and clarity of our work. We have conducted a comprehensive review and a more detailed description of the main concepts and variables used in our research, ensuring the clarity and logic of our research framework and results.

Reviewer #1:

First and foremost, we wish to express our sincerest gratitude to Reviewer #1 for their positive evaluation of our manuscript and for recognizing the depth and significance of our research.

Reviewer #4:

We also thank Reviewer #4 for their detailed and constructive feedback, which has played a significant role in improving our manuscript. Here are our responses to each of the points raised:

Literature on Digital Transformation Mechanisms: We have added two more recent and relevant literature references to strengthen the authority of these concepts. Thank you for pointing this out, which has made our theoretical framework more robust.

Abstract Semantics: The statement in the abstract has been revised to "the research indicates that digital transformation plays a key role in significantly enhancing supply chain efficiency," as suggested. This alteration better captures the essence of our findings and aligns with the empirical evidence presented.

Consistency in Citation Format: Following your suggestion, we have carefully reviewed and corrected the citation format throughout the manuscript, ensuring the consistency and accuracy of all references according to the prescribed guidelines. This includes a meticulous check of author names, publication years, and paper titles.

Organizational Structure: We have reorganized the section discussing the impact of digital transformation across different corporate backgrounds, implementing clear subheadings to differentiate various aspects. This structure provides better clarity and logic, enhancing the reading experience.

Standardization of Figures and Images: All figures and image annotations have been carefully checked and standardized to ensure the uniformity of the entire document. This standardization enhances the visual presentation and coherence of our research findings.

Concise Conclusion: The conclusion section has been revised to more concisely state the direct positive impact of digital transformation on company performance. The new phrasing, "digital transformation drives down future external transaction costs, thereby consolidating the enterprise's market competitiveness and elevating financial performance," succinctly encapsulates our core message and findings.

We hope our revisions and responses adequately address the concerns and comments raised by the reviewers.

Yours sincerely,

Min Fan
